# Supplementary material for: Mechanism of lipid droplet formation by the yeast Sei1/Ldb16 Seipin complex
Source: Nat Commun. 2021 Oct 8;12:5892. doi: 10.1038/s41467-021-26162-6 (PMC8501077; doi:10.1038/s41467-021-26162-6)
Supplement: Supplementary file 1 — Supplementary Informatio [file 41467_2021_26162_MOESM1_ESM.pdf]

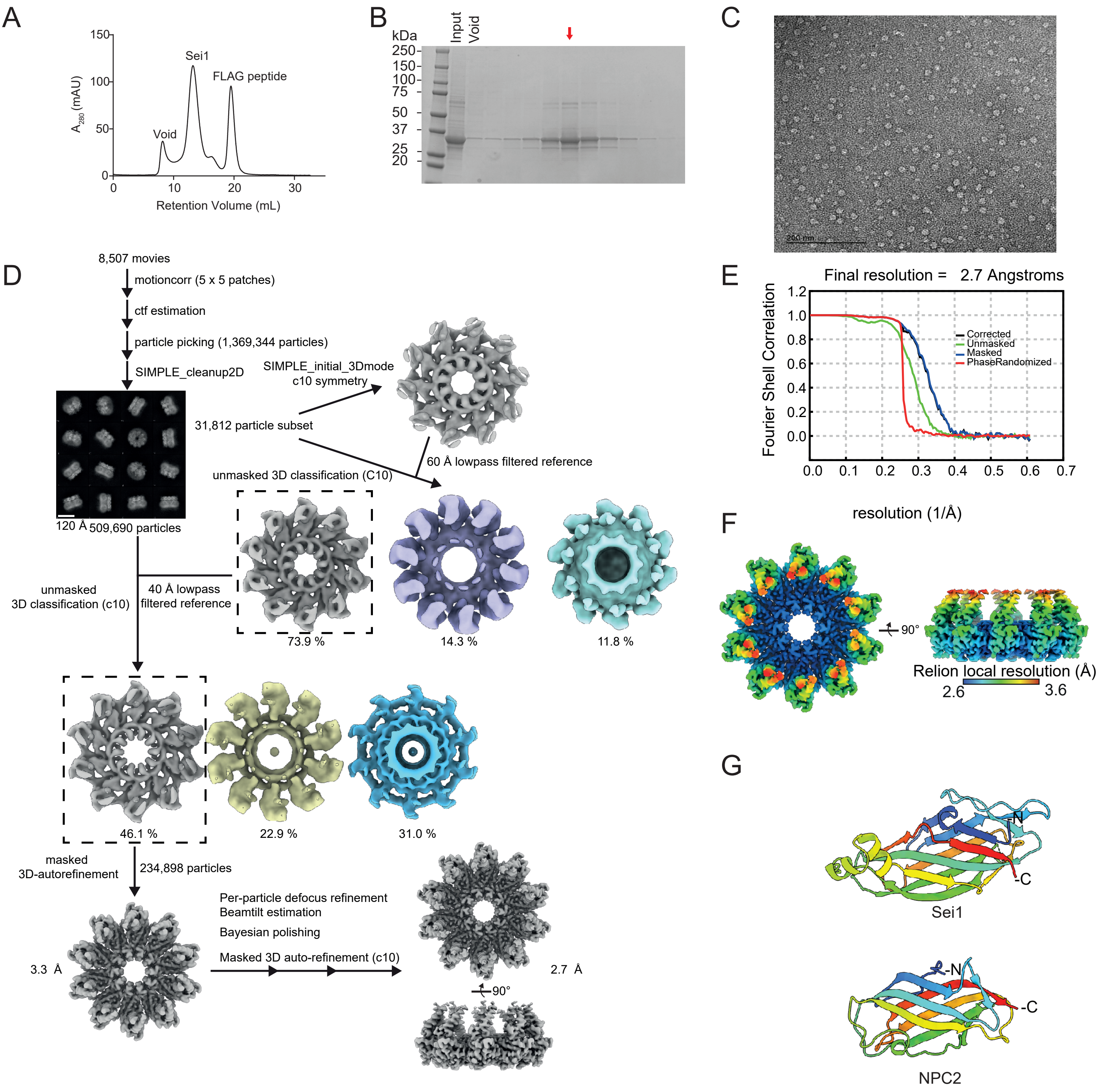
A
Sei1
FLAG peptide
Void
A280 (mAU)
Retention Volume (mL)
B
kDa
Input
Void
250
150
100
75
50
37
25
20
C
D
8,507 movies
motioncorr (5 x 5 patches)
ctf estimation
particle picking (1,369,344 particles)
SIMPLE_cleanup2D
SIMPLE_initial_3Dmode
c10 symmetry
31,812 particle subset
60 Å lowpass filtered reference
unmasked 3D classification (C10)
120 Å
509,690 particles
unmasked
3D classification (c10)
40 Å lowpass
filtered reference
73.9 %
14.3 %
11.8 %
46.1 %
22.9 %
31.0 %
masked
3D-autorefinement
234,898 particles
Per-particle defocus refinement
Beamtilt estimation
Bayesian polishing
Masked 3D auto-refinement (c10)
3.3 Å
2.7 Å
90°
E
Final resolution = 2.7 Angstroms
Fourier Shell Correlation
Corrected
Unmasked
Masked
PhaseRandomized
resolution (1/Å)
F
90°
Relion local resolution (Å)
2.6
3.6
G
N
C
Sei1
N
C
NPC2

Figure S1 - Structure determination of Sei1

(a) Size exclusion chromatogram of the Sei1-3xFLAG purification run used for cryo-EM structure determination. A_{280} is the absorbance at 280 nm in arbitrary units.

(b) Instant Blue-stained SDS PAGE gel of purified Sei1-3xFLAG. Input pertains to the material injected onto the size exclusion column. Each lane is taken from a consecutive 1mL fraction following the void fraction. Red arrow denotes sample taken for cryo-EM structure determination. At least 3 repeats of the purification were carried out with similar results.

(c) Representative negative stain of purified Sei1-3xFLAG used for structural analysis. Negative stain was carried at twice with similar results.

(d) Cryo-EM data processing workflow for Sei1.

(e) Gold-standard FSC curves used for global-resolution estimates of Sei1 map, as determined within RELION-3.1. Red, phase-randomized; green, unmasked; blue, masked; black, corrected.

(f) Local-resolution estimation of reconstructed Sei1 map as determined within RELION-3.1. Volume contoured at threshold level of 0.007, with detergent density omitted for clarity.

(g) Side by side depictions of the Sei1 luminal domain and NPC2 (PDB 2HKA.

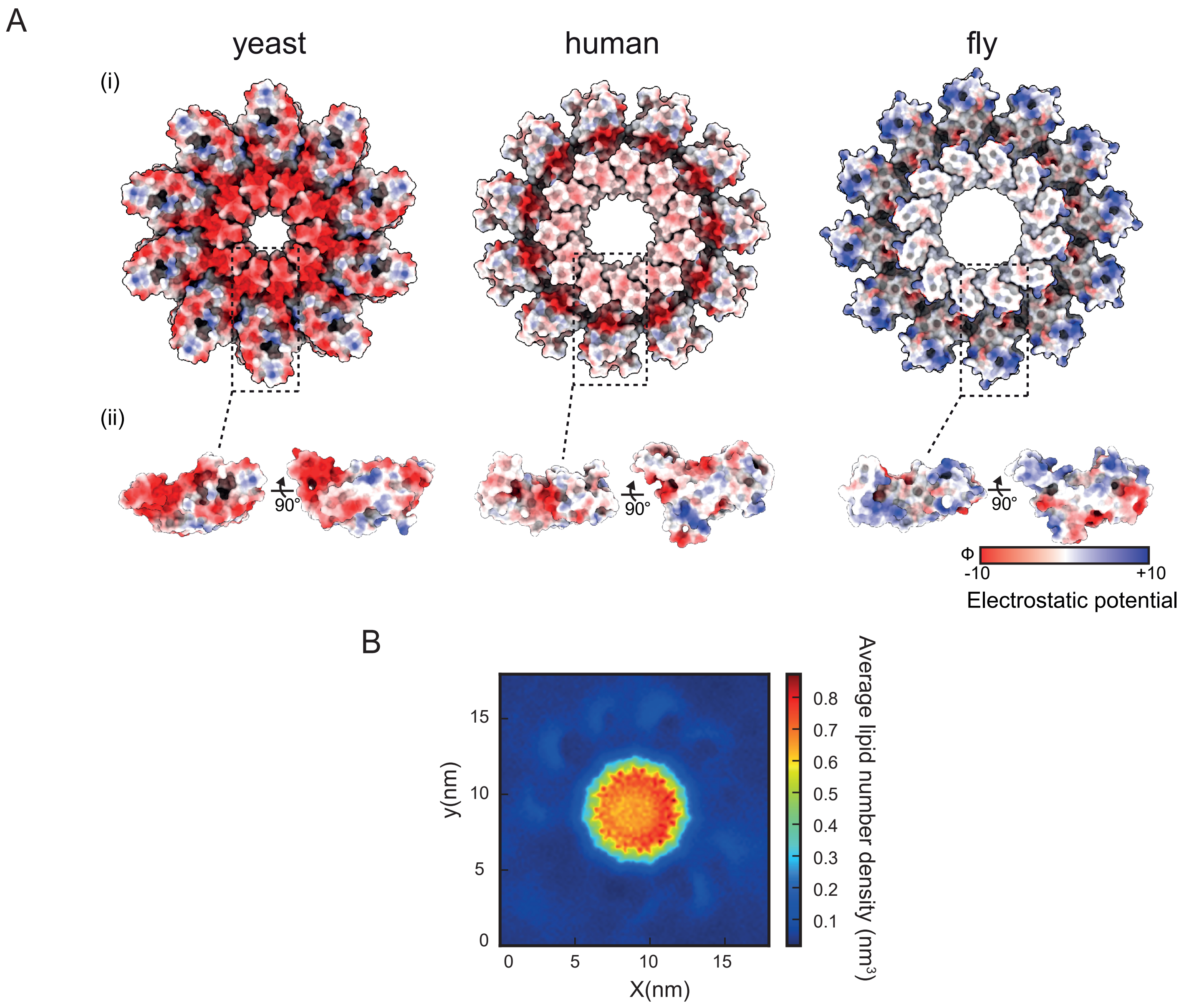
A
yeast
human
fly
(i)
(ii)
90°
90°
90°
Φ
-10
+10
Electrostatic potential
B
y(nm)
X(nm)
0
5
10
15
Average lipid number density (nm³)
0.1
0.2
0.3
0.4
0.5
0.6
0.7
0.8

Figure S2 – comparison of Seipin luminal domains

(a) Coulombic electrostatic potential surface representation (Red, negative charge; blue, positive charge; white, no charge) of Seipin luminal domains from yeast (left), human (middle) and fly (right), as determined in ChimeraX. (i) View of homodecameric assembly from cytosol or (ii) as individual protomers.

(b) Top View of coarse-grained molecular dynamic simulations of Human Seipin (PDB 6DS5) in a POPC membrane with 3% trioleoylglycerol. Images depict average lipid number density of trioleoylglycerol.

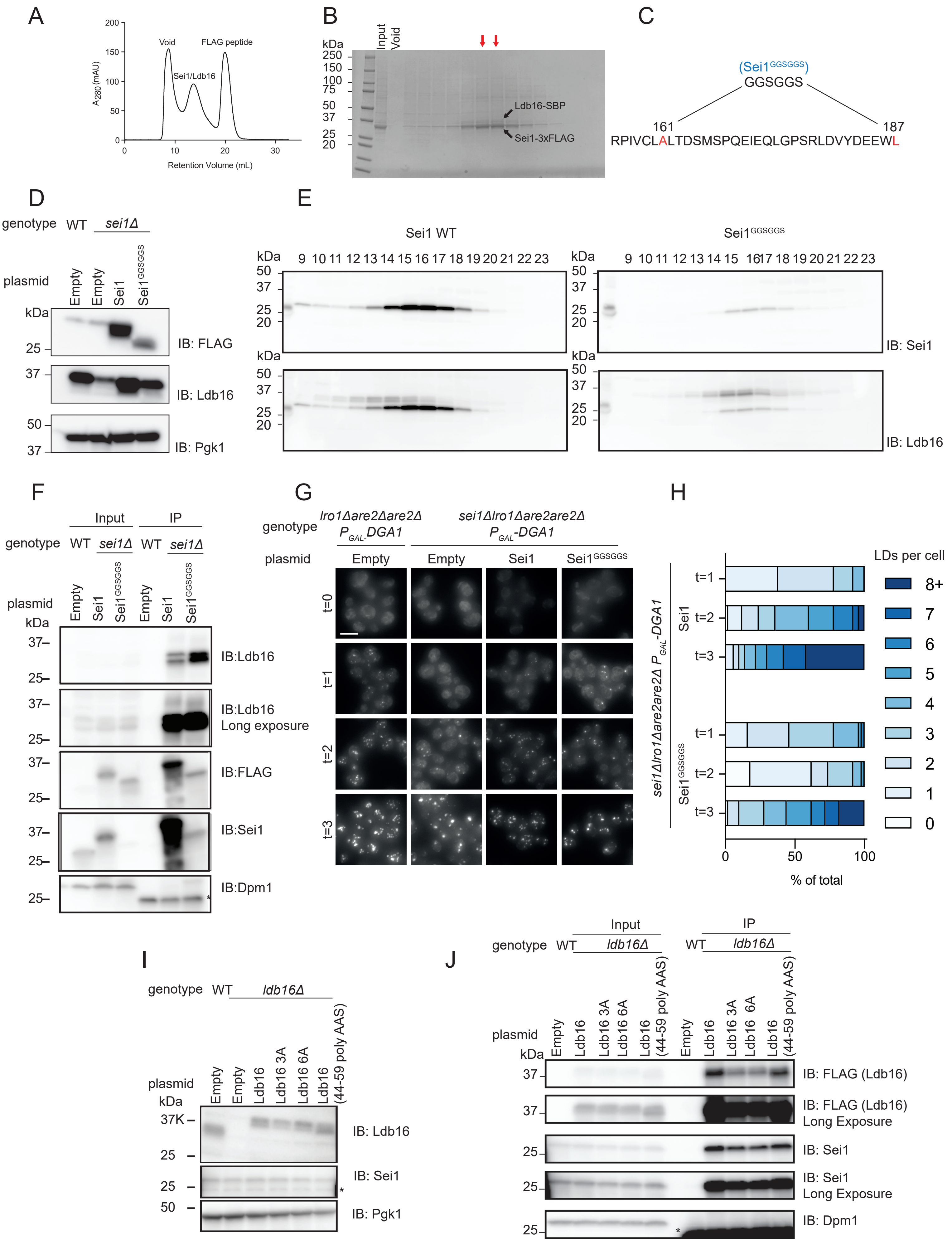
A
Void
FLAG peptide
Sei1/Ldb16
Retention Volume (mL)
B
Input
Void
Ldb16-SBP
Sei1-3xFLAG
C
(Sei1GGSGGS)
GGSGGS
161
187
RPIVCLALTDSMSPQEIEQLGPSRLDVYDEEWL
D
genotype WT sei1Δ
plasmid Empty Empty Sei1 Sei1GGSGGS
IB: FLAG
IB: Ldb16
IB: Pgk1
E
Sei1 WT
Sei1GGSGGS
IB: Sei1
IB: Ldb16
F
Input
IP
genotype WT sei1Δ WT sei1Δ
plasmid Empty Sei1 Sei1GGSGGS Empty Sei1 Sei1GGSGGS
IB:Ldb16
IB:Ldb16 Long exposure
IB:FLAG
IB:Sei1
IB:Dpm1
G
genotype lro1Δare2Δare2Δ PGAL-DGA1
sei1Δlro1Δare2are2Δ PGAL-DGA1
plasmid Empty Empty Sei1 Sei1GGSGGS
t=0
t=1
t=2
t=3
H
LDs per cell
8+
7
6
5
4
3
2
1
0
sei1Δlro1Δare2are2Δ PGAL-DGA1
Sei1
Sei1GGSGGS
% of total
I
genotype WT ldb16Δ
plasmid Empty Empty Ldb16 Ldb16 3A Ldb16 6A Ldb16 (44-59 poly AAS)
IB: Ldb16
IB: Sei1
IB: Pgk1
J
Input
IP
genotype WT ldb16Δ WT ldb16Δ
plasmid Empty Ldb16 Ldb16 3A Ldb16 6A Ldb16 (44-59 poly AAS) Empty Ldb16 Ldb16 3A Ldb16 6A Ldb16 (44-59 poly AAS)
IB: FLAG (Ldb16)
IB: FLAG (Ldb16) Long Exposure
IB: Sei1
IB: Sei1 Long Exposure
IB: Dpm1

Figure S3 – Ldb16 complements Sei1 structure for LD formation

(a) Size exclusion chromatogram of the Sei1-3xFLAG and Ldb16-SBP purification run used for cryo-EM structure determination. A_{280} is the absorption at 280 nm in arbitrary units

(b) Instant Blue-stained SDS PAGE gel of co-purified Sei1-3xFLAG and Ldb16-SBP. Input pertains to the material injected onto the size exclusion column. Each lane is taken from a consecutive 1ml fraction following the void fraction. Red arrows denote samples taken for cryo-EM structure determination. Two repeats of the purification were carried out with similar results.

(c) Sei1 sequence of the central helices region and the segment replaced by GGSGGS linker (residues 161-187)

(d) Western blot of Sei1 and Ldb16 in sei1Δ cells endogenously expressing Sei1 WT and Sei1GGSGGS from a plasmid. This was repeated three times with similar results.

(e) Immuno blots following size exclusion chromatography of 1% DDM 0.01% CHS solubilized *sei1Δ* cells, expressing either WT Sei1 or $Sei1^{GGSGGS}$ from a plasmid under an *ADH1* promoter. A sample of fractions 9-23 (1ml each) from a superose6 column were run on an SDS PAGE gel and immunoblotted for Sei1 and Ldb16. This was repeated twice with similar results.

(f) WT Sei1, WT Sei1 FLAG and Sei^{GGSGGS}-FLAG were immunoprecipitated with FLAG beads from a crude membrane fraction solubilized with 1% DMNG. Eluted proteins were analyzed by SDS–PAGE and immunoblotting. *=Non specific bands. This was repeated twice with similar results.

(g) Analysis of LDs in cells with the indicated genotype after staining with the neutral lipid dye BODIPY 493/503. Lipid droplet biogenesis was induced by the addition of 2% galactose to cells with indicted genotype expressing DGA1 from the *GAL1* promoter. Time is in hours. Scale bar corresponds to 5 μm.

(h) Quantification of LD/cell upon induction of LD formation as in (g). Number of LDs per cell was quantified for a minimum of 50 cells per timepoint.

(i) Western blot of Sei1 and Ldb16 in cells with the indicated genotype. Proteins were expressed at endogenous levels.

(j) Ldb16-FLAG and FLAG tagged Ldb16 mutants were immunoprecipitated with FLAG beads from a crude membrane fraction solubilized with 1% DMNG. Eluted proteins were analyzed by SDS–PAGE and immunoblotting. *=Nonspecific bands.

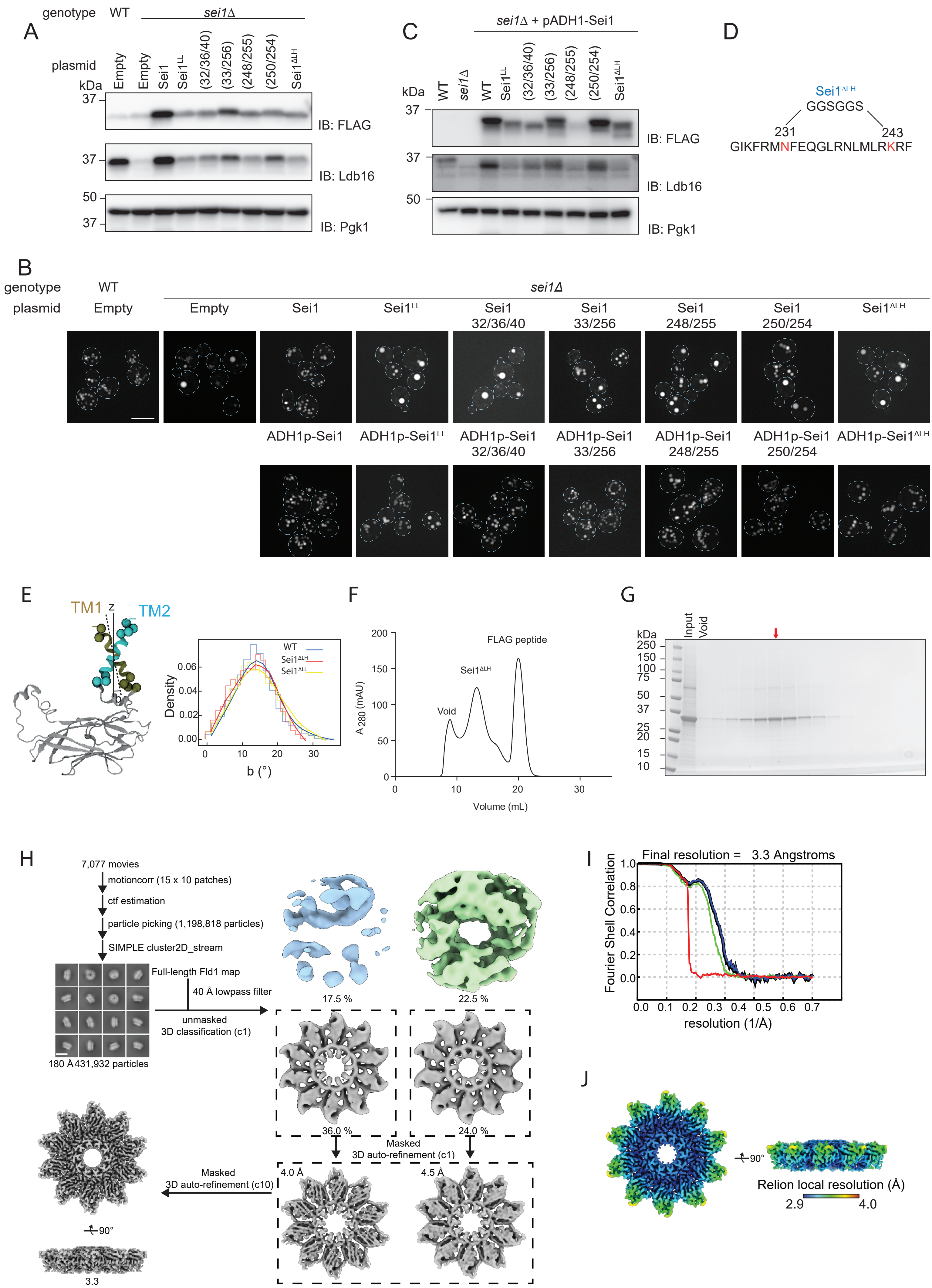
A
genotype WT sei1Δ
plasmid Empty Empty Sei1 Sei1LL (32/36/40) (33/256) (248/255) (250/254) Sei1ΔLH
kDa
IB: FLAG
IB: Ldb16
IB: Pgk1
B
genotype WT sei1Δ
plasmid Empty Empty Sei1 Sei1LL Sei1 32/36/40 Sei1 33/256 Sei1 248/255 Sei1 250/254 Sei1ΔLH
ADH1p-Sei1 ADH1p-Sei1LL ADH1p-Sei1 32/36/40 ADH1p-Sei1 33/256 ADH1p-Sei1 248/255 ADH1p-Sei1 250/254 ADH1p-Sei1ΔLH
C
sei1Δ + pADH1-Sei1
WT sei1Δ WT Sei1LL (32/36/40) (33/256) (248/255) (250/254) Sei1ΔLH
IB: FLAG
IB: Ldb16
IB: Pgk1
D
Sei1ΔLH
GGSGGS
231 243
GIKFRMNFEQGLRNLMLRKRF
E
TM1 TM2
z
b
Density
b (°)
WT
Sei1ΔLH
Sei1ΔLL
F
FLAG peptide
Sei1ΔLH
Void
A280 (mAU)
Volume (mL)
G
kDa
Input
Void
H
7,077 movies
motioncorr (15 x 10 patches)
ctf estimation
particle picking (1,198,818 particles)
SIMPLE cluster2D_stream
Full-length Fld1 map
40 Å lowpass filter
unmasked
3D classification (c1)
180 Å 431,932 particles
17.5 %
22.5 %
36.0 %
24.0 %
Masked
3D auto-refinement (c1)
4.0 Å
4.5 Å
Masked
3D auto-refinement (c10)
90°
3.3
I
Final resolution = 3.3 Angstroms
Fourier Shell Correlation
resolution (1/Å)
J
90°
Relion local resolution (Å)
2.9 4.0

Figure S4 – Sei1 Locking Helix positions transmembrane segments and facilitates Ldb16 binding

(a) Western blot of Sei1 and Ldb16 in cells with the indicated genotype. Proteins were expressed at endogenous levels. Indicated residues were replaced with alanine unless stated otherwise. This was repeated twice with similar results.

(b) Analysis of LDs in cells with the indicated genotype after staining with the neutral lipid dye Bodipy493/503. Scale bar corresponds to 5 µm. Indicated residues were replaced with alanine unless stated otherwise. A minimum of 3 biological repeats were analyzed with similar results.

(c) Western blot of Sei1 and Ldb16 in cells with the indicated genotype. Proteins were expressed under the *ADH1* promoter. Indicated residues were replaced with alanine unless stated otherwise. This was repeated twice with similar results.

(d) Sei1 sequence of the LH and the segment replaced by GGSGGS linker (residues 231-243)

(e) schematic representation of the angle (a) between Sei1 TM1 and TM2 analyzed by atomistic molecular dynamic simulations. Data are plotted as per Fig 4E and F.

(f) Size exclusion chromatogram of the Sei1$^{\Delta LH}$ purification run used for cryo-EM structure determination. A_{280} is the absorption at 280 nm in arbitrary units.

(g) Instant Blue-stained SDS PAGE gel of purified Sei1 $^{\Delta LH}$. Input pertains to the material injected onto the size exclusion column. Each lane is taken from a consecutive 1ml fraction following the void fraction. Red arrow denotes sample taken for cryo-EM structure determination.

(h) Cryo-EM data processing workflow for Sei1$^{\Delta LH}$.

(i) Gold-standard FSC curves used for global-resolution estimates of Sei1$^{\Delta LH}$ map, as determined within RELION-3.1. Red, phase-randomized; green, unmasked; blue, masked; black, corrected.

(j) Local-resolution estimation of reconstructed Sei1$^{\Delta LH}$ map as determined within RELION-3.1. Volume contoured at threshold level of 0.04, with detergent density omitted for clarity.

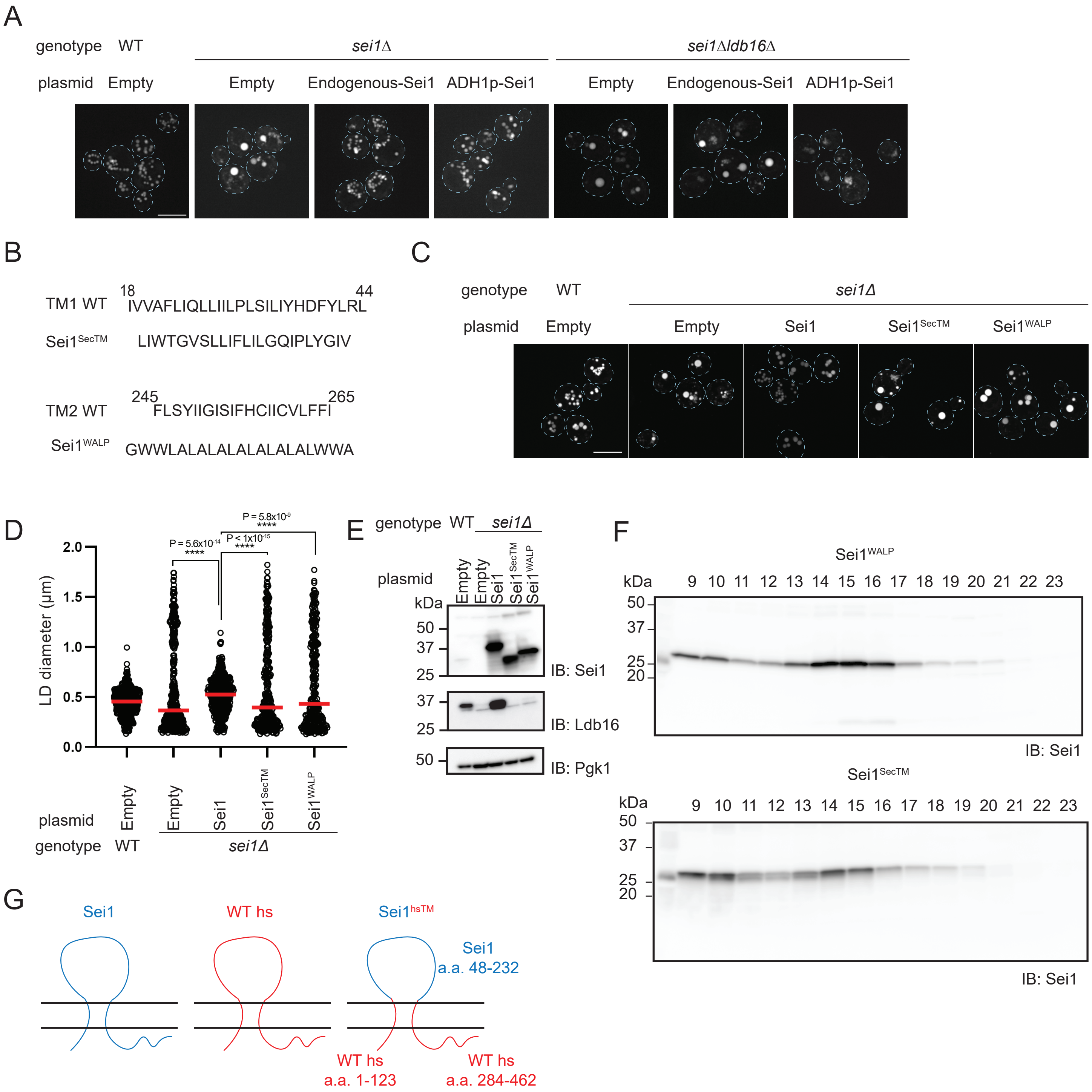

A
genotype WT
sei1Δ
sei1Δldb16Δ
plasmid Empty
Empty
Endogenous-Sei1
ADH1p-Sei1
Empty
Endogenous-Sei1
ADH1p-Sei1
B
TM1 WT 18 IVVAFLIQLLIILPLSILIYHDFYLRL 44
Sei1SecTM LIWTGVSLLIFLILGQIPLYGIV
TM2 WT 245 FLSYIIGISIFHCIICVLFFI 265
Sei1WALP GWWLALALALALALALWWA
C
genotype WT
sei1Δ
plasmid Empty
Empty
Sei1
Sei1SecTM
Sei1WALP
D
P = 5.8x10-9
****
P = 5.6x10-14
****
P < 1x10-15
****
LD diameter (µm)
2.0
1.5
1.0
0.5
0.0
plasmid
Empty
Empty
Sei1
Sei1SecTM
Sei1WALP
genotype WT
sei1Δ
E
genotype WT sei1Δ
plasmid
Empty
Empty
Sei1
Sei1SecTM
Sei1WALP
kDa
50
37
25
IB: Sei1
37
25
IB: Ldb16
50
IB: Pgk1
F
Sei1WALP
kDa
9 10 11 12 13 14 15 16 17 18 19 20 21 22 23
50
37
25
20
IB: Sei1
Sei1SecTM
kDa
9 10 11 12 13 14 15 16 17 18 19 20 21 22 23
50
37
25
20
IB: Sei1
G
Sei1
WT hs
Sei1hsTM
Sei1
a.a. 48-232
WT hs
a.a. 1-123
WT hs
a.a. 284-462

Figure S5 – Sei1 transmembrane segments contribute to LD formation

(a) Analysis of LDs in cells with the indicated genotype after staining with the neutral lipid dye Bodipy493/503. Scale bar corresponds to 5 µm. A minimum of 3 biological repeats were analyzed with similar results.

(b) Amino acid sequence of Sec61 TM1 and WALPs used to replace TM1 ($Sei1^{SecTM}$) and TM2 ($Sei1^{WALP}$), respectively.

(c) Analysis of LDs in cells with the indicated genotype after staining with the neutral lipid dye Bodipy493/503. Scale bar corresponds to 5 µm.

(d) Quantification of LD diameter of cells shown in (b). At least 100 LDs were analysed for a minimum of 3 biological repeats. Red bars represent median diameter. n=3. Difference in distribution of LD size was tested using a two sided Kolmogorov-Smirnov test (**** $p < 0.0001$, *n.s.* non-significant).

(e) Western blot of Sei1 and Ldb16 in cells with the indicated genotype. Proteins were expressed under the *ADH1* promoter. This was repeated three times with similar results.

(f) Immuno blots following size exclusion chromatography of 1% DDM 0.01% CHS solubilized *sei1Δ* cells, expressing either $Sei1^{WALP}$ or $Sei1^{SecTM}$ from a plasmid under an *ADH1* promoter. A sample of fractions 9-23 (1ml each) from a superose6 column were run on an SDS PAGE gel and immunoblotted for Sei1. This was repeated twice with similar results.

(h) Scheme of $Sei1^{hsTM}$. Sei1 -blue, WT hs -red.

Table S1 - List of strains used in this study

Strain	Identifier	Genotype	Origin
**BY4741**	yPC1505	MATa *his3Δ1 leu2Δ0 met15Δ0 ura3Δ0*	(Brachmann et al., 1998)
**FY251**	yPC1507	MATa *his3Δ1 leu2Δ0 trp1Δ63 ura3-52*	Fred Winston
sei1Δ	yPC3975	MATa *his3Δ1 leu2Δ0 met15Δ0 ura3Δ0 sei1::KanR*	(Grippa et al., 2015)
ldb16Δ	yPC4281	MATa *his3Δ1 leu2Δ0 met15Δ0 ura3Δ0 ldb16::NAT*	This study
sei1Δ ldb16Δ	yPC4299	MATa *his3Δ1 leu2Δ0 met15Δ0 ura3Δ0 sei1::NAT ldb16::hygB*	This study
sei1Δ Ldb16-HA	yPC12392	MATa *his3Δ1 leu2Δ0 trp1Δ63 ura3-52 sei1::NAT LDB16-3xHA-HIS*	This study

Table S2 - List of plasmids used in this study

Identifier	Plasmid	Origin
**bPC557**	pESC-TRP *TyrRS-tRNA CUA* for pBpa incorporation	(Chin et al., 2003)
**bPC1940**	pRS416 P_{ADH1} *Sei1-3xFLAG* T_{ADH1}	This study
**bPC1887**	pRS423 P_{GAL1} *Sei1-3xFLAG* T_{CYC1}	This study
**bPC1888**	pRS426 P_{GAL1} *LDB16-SBP* T_{CYC1}	This study
**bPC1978**	pRS423 P_{GAL1} *Sei1(231-243 to GGSGGS)-3xFLAG* T_{CYC1}	This study
**bPC2042**	pRS416 P_{SEI1} *Sei1-3xFLAG* T_{ADH1}	This study
**bPC2043**	pRS416 P_{SEI1} *Sei1(231-243 to GGSGGS)-3xFLAG* T_{ADH1}	This study
**bPC2045**	pRS416 P_{SEI1} *Sei1 (161-187 to GGSGGS)-3xFLAG* T_{ADH1}	This study
**bPC2046**	pRS416 P_{SEI1} *Sei1 (Y37L Y41L)-3xFLAG* T_{ADH1}	This study
**bPC2047**	pRS416 P_{SEI1} *Sei1 (Y248L F255L)-3xFLAG* T_{ADH1}	This study
**bPC2048**	pRS416 P_{SEI1} *Sei1 (S33A H256A)-3xFLAG* T_{ADH1}	This study
**bPC2053**	pRS416 P_{SEI1} *Sei1 (I250A I254A)-3xFLAG* T_{ADH1}	This study
**bPC2113**	pRS416 P_{SEI1} *Sei1 (L32A I36A F40A)-3xFLAG* T_{ADH1}	This study
**bPC2040**	pRS416 P_{ADH1} *Sei1(231-243 to GGSGGS)-3xFLAG* T_{ADH1}	This study
**bPC2041**	pRS416 P_{ADH1} *Sei1 (Y37L Y41L)-3xFLAG* T_{ADH1}	This study
**bPC2105**	pRS416 P_{ADH1} *Sei1 (Y248L F255L)-3xFLAG* T_{ADH1}	This study
**bPC2106**	pRS416 P_{ADH1} *Sei1 (S33A H256A)-3xFLAG* T_{ADH1}	This study
**bPC2109**	pRS416 P_{ADH1} *Sei1 (I250A I254A)-3xFLAG* T_{ADH1}	This study
**bPC2119**	pRS416 P_{ADH1} *Sei1 (L32A I36A F40A)-3xFLAG* T_{ADH1}	This study
**bPC2124**	pRS316 P_{LDB16} *LDB16-3xFLAG* T_{ADH1}	This study
**bPC2127**	pRS316 P_{LDB16} *LDB16 (S53A/T54A/S55A)-3xFLAG* T_{ADH1}	This study

**bPC2130**	pRS316 *P_{LDB16} LDB16 (S53/55/62A T52/61/63A)-3xFLAG T_{ADH1}*	This study
**bPC2131**	pRS316 *P_{LDB16} LDB16 (44-59 to AASAAS)-3xFLAG T_{ADH1}*	This study
**bPC2132**	pRS416 *P_{ADH1} hs_BSCL2_isoform3-3xFLAG T_{ADH1}*	This study
**bPC2132**	pRS416 *P_{ADH1} BSCL2iso3(1-123)-Sei1(48-232)-BSCL2iso3(284-462)-3xFLAG T_{ADH1}*	This study
**bPC2083**	pRS416 *P_{ADH1} Sei1-L26 amber (TAG)-3xFLAG T_{ADH1}*	This study
**bPC2084**	pRS416 *P_{ADH1} Sei1-I29 amber (TAG)-3xFLAG T_{ADH1}*	This study
**bPC2085**	pRS416 *P_{ADH1} Sei1-L30 amber (TAG)-3xFLAG T_{ADH1}*	This study
**bPC2086**	pRS416 *P_{ADH1} Sei1-H38 amber (TAG)-3xFLAG T_{ADH1}*	This study
**bPC2087**	pRS416 *P_{ADH1} Sei1-F40 amber (TAG)-3xFLAG T_{ADH1}*	This study
**bPC2088**	pRS416 *P_{ADH1} Sei1-L162 amber (TAG)-3xFLAG T_{ADH1}*	This study
**bPC2089**	pRS416 *P_{ADH1} Sei1-T163 amber (TAG)-3xFLAG T_{ADH1}*	This study
**bPC2090**	pRS416 *P_{ADH1} Sei1-S165 amber (TAG)-3xFLAG T_{ADH1}*	This study
**bPC2091**	pRS416 *P_{ADH1} Sei1-P168 amber (TAG)-3xFLAG T_{ADH1}*	This study
**bPC2092**	pRS416 *P_{ADH1} Sei1-Q169 amber (TAG)-3xFLAG T_{ADH1}*	This study
**bPC2093**	pRS416 *P_{ADH1} Sei1-E170 amber (TAG)-3xFLAG T_{ADH1}*	This study
**bPC2094**	pRS416 *P_{ADH1} Sei1-E172 amber (TAG)-3xFLAG T_{ADH1}*	This study
**bPC2095**	pRS416 *P_{ADH1} Sei1-P176 amber (TAG)-3xFLAG T_{ADH1}*	This study
**bPC2096**	pRS416 *P_{ADH1} Sei1-W186 amber (TAG)-3xFLAG T_{ADH1}*	This study
**bPC2097**	pRS416 *P_{ADH1} Sei1-L187 amber (TAG)-3xFLAG T_{ADH1}*	This study
**bPC2098**	pRS416 *P_{ADH1} Sei1-F245 amber (TAG)-3xFLAG T_{ADH1}*	This study

**bPC2099**	pRS416 P_{ADH1} *Sei1-L246 amber (TAG)-3xFLAG* T_{ADH1}	This study
**bPC2100**	pRS416 P_{ADH1} *Sei1-I252 amber (TAG)-3xFLAG* T_{ADH1}	This study
**bPC2101**	pRS416 P_{ADH1} *Sei1-I254 amber (TAG)-3xFLAG* T_{ADH1}	This study
**bPC2102**	pRS416 P_{ADH1} *Sei1-I259 amber (TAG)-3xFLAG* T_{ADH1}	This study
**bPC2103**	pRS416 P_{ADH1} *Sei1-F263 amber (TAG)-3xFLAG* T_{ADH1}	This study

Table S3. Oligonucleotides used in this study.

Primer	Nucleotide sequence (5′–3′)	Purpose
185	CTATTGTACTCGAGCGAGGCAAGCTAAACAGATC	Reverse Primer 50 bp downstream ADH1 terminator
491	CGGACATCGTTAATATAAAGATTTTACGAAGGAATTCTAGGGGTCGACGGATCCCCGGGTT	Forward primer for tagging of LDB16 ORF
492	TCTATCATTCACTTGTTAGTGCATGAGAAGAAGTAATTGCTCGATGAATTCGAGCTCGTT	Reverse primer for tagging/deletion of LDB16 ORF
570	GCAACTGTAGGAGGAGAAAGCAGGTATATAACTAGCCGCAATCGGATCCCCGGGTTAATTAA	Forward primer for deletion of LDB16 ORF
763	CTTAATGATTGCGGCCGCTGATAGACAACCACACGGTC	Forward Primer 450 bp upstream of LDB16 ORF
4037	CAATCAACTTCCGGAGTGTAAAAACTGATTTTCAATGTCTACAGAAAAGGTAGACC	Forward primer for insertion of BSCL2 into pRS416-ADH1P
4038	TTATTTAGAAGTGGCGCGCCTCAGGAACTAAAGCAGGGGG	Reverse primer for insertion of BSCL2 into pRS416-ADH1P
4043	ACTATTCCTATATGCCGACAGATTCCTCTAACGTAGTCCC	Forward Primer amplifying centre of Sei1 for construction of BSCL2-Sei1 chimeras
4044	AGGTATCTGAGCCCAGTGAAAAAATTCATCCTAAACTTAATCCCACT	Reverse Primer amplifying centre of Sei1 for construction of BSCL2-Sei1 chimeras
4041	GCTGAGGTGGCTGACGGCAGGTAATAGTCTTAGGTAA	Reverse Primer amplifying C-terminal part of BSCL2 for construction of BSCL2-Sei1 chimeras
4042	CGCATCCACGCGCACGAGCAGGGATTAAGAAACTT	Forward Primer amplifying N-terminal part of BSCL2 for construction of BSCL2-Sei1 chimeras
4093	GATTCTAGAACTAGTATGAAAATCAATGTATCCCGTCCATT	Amplifying Sei1 3xFlag from pRS423 PGAL1 Forward
4094	TACATGACTCGAGCTACTTGTCATCGTCATCCTTGTAGTCG	Amplifying Sei1 3xFlag from pRS423 PGAL1 Reverse
4095	GAACTAGTGGATCCATGTTTGTGGTGGATTGGAGC	Amplifiying ldb16-SBP from pRS426 PGAL1 Forward
4096	CATGACTCGAGGTCGACTCATGGTTCACGTTGACC	Amplifiying ldb16-SBP from pRS426 PGAL1 Reverse
4107	GAAAGTGGGATTAAGTTTAGGATGGGTGGCAGTGGTGGCAGTAGATTTTTATCTTATATTATTGGC	Generating Sei1 N231-K243 replaced with GGSGGS linker Forward

4108	GCCAATAATATAAGATAAAAATCTACTGCCACCAC TGCCACCCATCCTAAACTTAATCCCACTTTC	Generating Sei1 N231-K243 replaced with GGSGGS linker Reverse
4109	ACTGCCACCACTGCCACCGAGGCAGACAATAGGTCT AGAAG	Generating Sei1 A161-G175/L187 replaced with GGSGGS linker Reverse
4110	GGTGGCAGTGGTGGCAGTCCATCACGTCTAGACGT TTAC	Generating Sei1 A161-G175 replaced with GGSGGS linker Forward
4111	GGTGGCAGTGGTGGCAGTAATACAATAAGAATAGA GGACAAAATATC	Generating Sei1 A161-L187 replaced with GGSGGS linker Forward
4118	CAATCAACTTCCGGAGTGTAAAAACTGATTTTCAA TGAAAATCAATGTATCCCG	Forward primer for amplifying Sei1 from pRS416 plasmid
4120	CAGGAAAAATCCAAGAAACATAGCTGAGGCGCGCC ACTTCTAAATAA	Reverse primer for amplifying Sei1 from pRS416 plasmid
4159	CATTCTTCCTTTATCGATCTTAATATTACACGATTT TTTACTAAGACTATTACCTGCC	Generating Y37L Y41L (Sei1ΔLL) point mutations into Sei1 Forward
4160	GGCAGGTAATAGTCTTAGTAAAAAATCGTGTAATA TTAAGATCGATAAAGGAAGAATG	Generating Y37L Y41L (Sei1ΔLL) point mutations into Sei1 Reverse
4161	GCTTCGAAAAAGATTTTTATCTTTAATTATTGGCA TTTCAATTTTACATTGCATAATATGTGTAC	Generating Y248L F255L point mutations into Sei1 Forward
4162	GTACACATATTATGCAATGTAAAATTGAAATGCCA ATAATTAAAGATAAAAATCTTTTTCGAAGC	Generating Y248L F255L point mutations into Sei1 Reverse
4171	GCTAATCATTCTTCCTTTAGCGATCTTAATATATC ACGATTTTTACC	Generating S33A point mutations into Sei1 Forward
4172	GGTAAAAATCGTGATATATTAAGATCGCTAAAGGA AGAATGATTAGC	Generating S33A point mutations into Sei1 Reverse
4173	CTTATATTATTGGCATTTCAATTTTCGCTTGCATA ATATGTGTACTTTTTTTTATC	Generating H256A point mutations into Sei1 Forward
4174	GATAAAAAAAAGTACACATATTATGCAAGCGAAAA TTGAAATGCCAATAATATAAG	Generating H256A point mutations into Sei1 Reverse
4185	GAAAAAGATTTTTATCTTATATTGCTGGCATTTCA GCTTTCCATTGCATAATATGTGTAC	Generating I250A I254A point mutations into Sei1 Forward

4186	GTACACATATTATGCAATGGAAAGCTGAAATGCCAGCAATATAAGATAAAAATCTTTTTC	Generating I250A I254A point mutations into Sei1 Reverse
4195	CAATTTTCCATTGCATAATATGTGTACTTTAGTTTATCACAGGTTGCACTGCATTC	Generating amber codon site in Sei1 F263 Forward
4196	GAATGCAGTGCAACCTGTGATAAACTAAAGTACACATATTATGCAATGGAAAATTG	Generating amber codon site in Sei1 F263 Reverse
4197	ACGGATTCCATGTCGCCTCAGTAGATCGAACAACTAGGCCCATCACGTCTAG	Generating amber codon site in Sei1 E170 Forward
4198	TGATGGGCCTAGTTGTTCGATCTACTGAGGCGACATGGAATCCGTCAGTGCG	Generating amber codon site in Sei1 E170 Reverse
4199	GATTCCATGTCGCCTCAGGAGATCTAGCAACTAGGCCCATCACGTCTAGAC	Generating amber codon site in Sei1 E172 Forward
4200	GTCTAGACGTGATGGGCCTAGTTGCTAGATCTCCTGAGGCGACATGGAATC	Generating amber codon site in Sei1 E172 Reverse
4201	ATCTTAATATATCACGATTAGTACCTAAGACTATTACCTGCCGATTC	Generating amber codon site in Sei1 F40 Forward
4202	GAATCGGCAGGTAATAGTCTTAGGTACTAATCGTGATATATTAAGATCGATAAAG	Generating amber codon site in Sei1 F40 Reverse
4203	GAAACTTGATGCTTCGAAAAAGATAGTTATCTTATATTATTGGCATTTC	Generating amber codon site in Sei1 F245 Forward
4204	GAAATGCCAATAATATAAGATAACTATCTTTTTCGAAGCATCAAGTTTC	Generating amber codon site in Sei1 F245 Reverse
4205	CTTCCTTTATCGATCTTAATATATTAGGATTTTTACCTAAGACTATTAC	Generating amber codon site in Sei1 H38 Forward
4206	CTTCCTTTATCGATCTTAATATATTAGGATTTTTACCTAAGACTATTAC	Generating amber codon site in Sei1 H38 Reverse
4207	GCATTTCTGATACAATTGCTAATCTAGCTTCCTTTATCGATCTTAATATATC	Generating amber codon site in Sei1 I29 Forward
4208	GATATATTAAGATCGATAAAGGAAGCTAGATTAGCAATTGTATCAGAAATGC	Generating amber codon site in Sei1 I29 Reverse
4209	GATTTTTATCTTATATTATTGGCTAGTCAATTTTCCATTGCATAATATGTGTAC	Generating amber codon site in Sei1 I252 Forward
4210	ACATATTATGCAATGGAAAATTGACTAGCCAATAATATAAGATAAAAATCTTTTTC	Generating amber codon site in Sei1 I252 Reverse
4211	GATTTTTATCTTATATTATTGGCATTTCATAGTTCCATTGCATAATATGTGTACTTTTTTTATC	Generating amber codon site in Sei1 I254 Forward
4212	GTACACATATTATGCAATGGAACTATGAAATGCCAATAATATAAGATAAAAATCTTTTTC	Generating amber codon site in Sei1 I254 Reverse

4213	GGCATTTCAATTTTCCATTGCATATAGTGTGTACT TTTTTTTATCACAGGTTGCAC	Generating amber codon site in Sei1 I259 Forward
4214	GTGCAACCTGTGATAAAAAAAAGTACACACTATAT GCAATGGAAAATTGAAATGCC	Generating amber codon site in Sei1 I259 Reverse
4215	GTTGCATTTCTGATACAATAGCTAATCATTCTTCCT TTATC	Generating amber codon site in Sei1 L26 Forward
4216	GATAAAGGAAGAATGATTAGCTATTGTATCAGAAA TGCAACAACAATATATG	Generating amber codon site in Sei1 L26 Reverse
4217	GCATTTCTGATACAATTGCTAATCATTTAGCCTTT ATCGATCTTAATATATC	Generating amber codon site in Sei1 L30 Forward
4218	GATATATTAAGATCGATAAAGGCTAAATGATTAGC AATTGTATCAGAAATGC	Generating amber codon site in Sei1 L30 Reverse
4219	CTATTGTCTGCCTCGCATAGACGGATTCCATGTCGC CTC	Generating amber codon site in Sei1 L162 Forward
4220	GACATGGAATCCGTCTATGCGAGGCAGACAATAGG TCTAG	Generating amber codon site in Sei1 L162 Reverse
4221	GATCGAACAACTAGGCCCATCACGTTAGGACGTTT ACGATGAAGAATGGC	Generating amber codon site in Sei1 L179 Forward
4222	GCCATTCTTCATCGTAAACGTCCTAACGTGATGGGC CTAGTTGTTCGATC	Generating amber codon site in Sei1 L179 Reverse
4223	CTAGACGTTTACGATGAAGAATGGTAGAATACAAT AAGAATAGAGGACAAAATATC	Generating amber codon site in Sei1 L187 Forward
4224	GTCCTCTATTCTTATTGTATTCTACCATTCTTCATC GTAAACGTCTAGACGTG	Generating amber codon site in Sei1 L187 Reverse
4225	GAAACTTGATGCTTCGAAAAAGATTTTAGTCTTAT ATTATTGGCATTTCAATTTTCC	Generating amber codon site in Sei1 L246 Forward
4226	GGAAAATTGAAATGCCAATAATATAAGACTAAAAT CTTTTTCGAAGCATCAAGTTTC	Generating amber codon site in Sei1 L246 Reverse
4227	CTCGCACTGACGGATTCCATGTCGTAGCAGGAGAT CGAACAACTAGGCCCATCAC	Generating amber codon site in Sei1 P168 Forward
4228	GTGATGGGCCTAGTTGTTCGATCTCCTGCTACGACA TGGAATCCGTCAGTGCGAG	Generating amber codon site in Sei1 P168 Reverse
4229	CAGGAGATCGAACAACTAGGCTAGTCACGTCTAGA CGTTTACGATG	Generating amber codon site in Sei1 P176 Forward
4230	CATCGTAAACGTCTAGACGTGACTAGCCTAGTTGT TCGATCTCCTG	Generating amber codon site in Sei1 P176 Reverse
4231	ACTGACGGATTCCATGTCGCCTTAGGAGATCGAAC AACTAGGCCCATCACGTC	Generating amber codon site in Sei1 Q169 Forward

4232	GACGTGATGGGCCTAGTTGTTCGATCTCCTAAGGC GACATGGAATCCGTCA	Generating amber codon site in Sei1 Q169 Reverse
4233	ACTGACGGATTAGATGTCGCCTCAGGAGATCGAAC	Generating amber codon site in Sei1 S165 Forward
4234	CTCCTGAGGCGACATCTAATCCGTCAGTGCGAGGCA GAC	Generating amber codon site in Sei1 S165 Reverse
4235	GTCTGCCTCGCACTGTAGGATTCCATGTCGCCTCAG GAG	Generating amber codon site in Sei1 T163 Forward
4236	AGGCGACATGGAATCCTACAGTGCGAGGCAGACAA TAGGTCTAG	Generating amber codon site in Sei1 T163 Reverse
4237	CTAGACGTTTACGATGAAGAATAGCTAAATACAAT AAGAATAGAGG	Generating amber codon site in Sei1 W186 Forward
4238	CCTCTATTCTTATTGTATTTAGCTATTCTTCATCGT AAACGTCTAGACG	Generating amber codon site in Sei1 W186 Reverse
4239	CTTATTTAGAAGTGGCGCGCCTCACTTGTCATCGTC ATCCTTGTAG	Generating Sei1 tagging with 3xFLAG in pRS416
4240	TTGATTTGGACTGGTGTTTCTTTGTTGATTTTTTT GATTTTGGGTCAAATTCCATTGTATGGTATTGTTT TACCTGCCGATTCCTCTAACG	Generating sec61 TM1 instead of the Sei1 TM1 Forward
4241	AACAATACCATACAATGGAATTTGACCCAAAATCA AAAAAATCAACAAAGAAACACCAGTCCAAATCAAA TATGAACTCCATTGTAAAAACTGTAATGG	Generating sec61 TM1 instead of the Sei1 TM1 Reverse
4242	AGCCCACCACAGTGCTAGGGCGAGAGCAAGCGCCA GTGCTAGGGCGAGAGCAAGCCACCAACCTCTTTTTC GAAGCATCAAGTTTCTTAATC	Generating WALP sequence instead of TM2 of Sei1 Reverse
4244	GGTTGGTGGCTTGCTCTCGCCCTAGCACTGGCGCTT GCTCTCGCCCTAGCACTGTGGTGGGCTACAGGTTGC ACTGCATTCATTTTTG	Generating WALP sequence instead of TM2 of Sei1 Forward
4279	GCCCCCCCTGCTTTAGTTCCGACTACAAAGACCATG ACGGT	Forward primer for BSCL2 tagging with 3xFLAG by Gibson
4297	TGCTGTTTGTGCAACTAAACGTGCCAGTGCCGTCCC CAGGAACAACC	Forward primer for generating Ldb16 S53A S55A S62A point mutations.
4298	GCACTGGCACGTTTAGTTGCACAAACAGCAACATT AGACTTCTATGTGGTTTT	Reverse primer for generating Ldb16 S53A S55A S62A point mutations.
4320	CGCTGCAGCTTGTGCAACTAAACGTGCCAGTGCAGC CCCCAGGAACAACCTCAAT	Forward primer for Ldb16 to replace TSLS 52-55 by AALA and TST 61-63 by AAA.

4321	CTGGCACGTTTAGTTGCACAAGCTGCAGCGTTAGA CTTCTATGTGGTTTTGACGT	Reverse primer for Ldb16 to replace TSLS 52-55 by AALA and TST 61-63 by AAA.
4300	TGAGGCCGCAGAAGCCGCACTGGCAGCGTTGATAG GCAATGCCACA	Forward primer for Ldb16 to replace residues 44-59 by AASAAS
4301	GCTGCCAGTGCGGCTTCTGCGGCCTCACAAACAAGC ACATTAGACTTCTAT	Reverse primer for Ldb16 to replace residues 44-59 by AASAAS
4297	TGCTGTTTGTGCAACTAAACGTGCCAGTGCCGTCCC CAGGAACAACC	Forward primer for generating Ldb16 S53A S55A S62A point mutations.
4306	CGCATCGTGATACGCTAAGATCGAAGCAGGAAGAA TGATTAGCAATT	Reverse primer for generating Sei1 32/36/40 3xA point mutations
4307	GCTTCGATCTTAGCGTATCACGATGCGTACCTAAG ACTATTACCTGC	Forward primer for generating Sei1 32/36/40 3xA point mutations

Table S4: Cryo-EM data collection, refinement and validation statistics

	Sei1 (EMDB-13103) (PDB ID 7OXP)	Sei1$^{\Delta LH}$ (EMDB-13104) (PDB ID 7OXR)
**Data collection and processing**		
Magnification	165,000	105,000
Voltage (kV)	300	300
Electron exposure (e–/Å^2)	48	59.1
Defocus range (μm)	-1.0 to -3.0	-1.0 to -3.0
Pixel size (Å)	0.822	0.832
Symmetry imposed	C10	C10
Initial particle images (no.)	1,369,344	1,198,818
Final particle images (no.)	234,898	260,532
Map resolution (Å)	2.7	3.3
FSC threshold	0.143	0.143
Map resolution range (Å)	2.6-3.6	2.9-4.0
**Refinement**		
Initial model used (PDB code)	none	7OXP
Model resolution (Å)	2.7	3.3
FSC threshold	0.143	0.143
Model resolution range (Å)	2.6-3.6	2.9-4.0
Map sharpening *B* factor (Å^2)	-69.6	-151
Model composition		
Non-hydrogen atoms	18730	13670
Protein residues	2300	1700
Ligands	0	0
B factors (Å^2)		
Protein	54.7	33.8
Ligand	0	0
R.m.s. deviations		
Bond lengths (Å)	0.003	0.003
Bond angles (°)	0.585	0.581
Validation		
MolProbity score	1.49	1.54
Clashscore	8.06	8.45
Poor rotamers (%)	0.00	0.00
Ramachandran plot		
Favored (%)	97.79	97.59
Allowed (%)	2.21	2.41
Disallowed (%)	0.00	0.00
