# Supplementary material for: Mechanism of lipid droplet formation by the yeast Sei1/Ldb16 Seipin complex
Source: Nat Commun. 2021 Oct 8;12:5892. doi: 10.1038/s41467-021-26162-6 (PMC8501077; doi:10.1038/s41467-021-26162-6)
Supplement: Supplementary file 3 — Reporting Summary [file 41467_2021_26162_MOESM3_ESM.pdf]

nature portfolio

Corresponding author(s): *Double-blind peer review submissions: write DBPR and your manuscript number here instead of author names.*

Last updated by author(s): Jun 30, 2021

Reporting Summary

Nature Portfolio wishes to improve the reproducibility of the work that we publish. This form provides structure for consistency and transparency in reporting. For further information on Nature Portfolio policies, see our Editorial Policies and the Editorial Policy Checklist.

Statistics

For all statistical analyses, confirm that the following items are present in the figure legend, table legend, main text, or Methods section.

n/a	Confirmed	
☐	☒	The exact sample size (*n*) for each experimental group/condition, given as a discrete number and unit of measurement
☐	☒	A statement on whether measurements were taken from distinct samples or whether the same sample was measured repeatedly
☐	☒	The statistical test(s) used AND whether they are one- or two-sided<br>*Only common tests should be described solely by name; describe more complex techniques in the Methods section.*
☒	☐	A description of all covariates tested
☒	☐	A description of any assumptions or corrections, such as tests of normality and adjustment for multiple comparisons
☐	☒	A full description of the statistical parameters including central tendency (e.g. means) or other basic estimates (e.g. regression coefficient) AND variation (e.g. standard deviation) or associated estimates of uncertainty (e.g. confidence intervals)
☐	☒	For null hypothesis testing, the test statistic (e.g. *F*, *t*, *r*) with confidence intervals, effect sizes, degrees of freedom and *P* value noted<br>*Give P values as exact values whenever suitable.*
☒	☐	For Bayesian analysis, information on the choice of priors and Markov chain Monte Carlo settings
☒	☐	For hierarchical and complex designs, identification of the appropriate level for tests and full reporting of outcomes
☒	☐	Estimates of effect sizes (e.g. Cohen's *d*, Pearson's *r*), indicating how they were calculated

Our web collection on statistics for biologists contains articles on many of the points above.

Software and code

Policy information about availability of computer code

Data collection	Thermofisher EPU used to collect data on microscope and Gromacs 2019.4 for molecular dynamics data
Data analysis	SIMPLE3.0 - published and freely available<br>RELION3.1 - published and freely available<br>PHENIX 1.19.1_4122 - published and freely available<br>COOT 0.9.4.1 EL - published and freely available<br>ChimeraX 1.1.1 - published and freely available<br>PyMOL v.2.4.0 - published and freely available<br>Gromacs 2019.4 - published and freely available<br>VMD 1.9.4 - published and freely available<br>Matplotlib 3.4.2 - published and freely available<br>NumPy 1.16.2- published and freely available

For manuscripts utilizing custom algorithms or software that are central to the research but not yet described in published literature, software must be made available to editors and reviewers. We strongly encourage code deposition in a community repository (e.g. GitHub). See the Nature Portfolio guidelines for submitting code & software for further information.

Data

Policy information about availability of data

All manuscripts must include a data availability statement. This statement should provide the following information, where applicable:

- Accession codes, unique identifiers, or web links for publicly available datasets
- A description of any restrictions on data availability
- For clinical datasets or third party data, please ensure that the statement adheres to our policy

Coordinates for the structures have been deposited in the Protein Data Bank under accession codes PDB 7OXP (Sei1) and 7OXR (Sei1ΔLH). The electron microscopy volumes have been deposited in the Electron Microscopy Data Bank under accession codes EMD-13103 (Sei1) and EMD-13104 (Sei1ΔLH).
The following structures were used form the Protein Data Bank - PDB 6MLU (fly seipin) and PDB 6DS5 (human Seipin).

Field-specific reporting

Please select the one below that is the best fit for your research. If you are not sure, read the appropriate sections before making your selection.

☒ Life sciences ☐ Behavioural & social sciences ☐ Ecological, evolutionary & environmental sciences

For a reference copy of the document with all sections, see nature.com/documents/nr-reporting-summary-flat.pdf

Life sciences study design

All studies must disclose on these points even when the disclosure is negative.

Sample size	Sample size of lipid droplets was calculated by ImageJ. LDs have been shown to be homogeneous in size in wild type S. cerevisiae and therefore a minimum sample size of 100 LDs was chosen in order to achieve a comprehensive distribution.
Data exclusions	During lipid droplet sampling, a cut of of >XXX circularity was chosen. This leads to a loss in fluorescent marked events but leads to accurate depecition of LD diameter due to their circular nature.
Replication	All assays observing lipid droplet phenotype where replicated by at least three biological repeats. Biochemical experiments were replicated to verify reproducibility. No attempts at replication failed.
Randomization	For the observation of lipid droplet assays in yeast, each biological repeat represents a single unique clone that was chosen randomly following plasmid transformation. Randomization is not relevant to biochemical analyses used in this study as samples are not allocated into experimental groups.
Blinding	Blinding was not relevant in this study as the phenotypes observed were calculated via software with predetermined values.

Reporting for specific materials, systems and methods

We require information from authors about some types of materials, experimental systems and methods used in many studies. Here, indicate whether each material, system or method listed is relevant to your study. If you are not sure if a list item applies to your research, read the appropriate section before selecting a response.

Materials & experimental systems

n/a	Involved in the study	
☐	☒	Antibodies
☒	☐	Eukaryotic cell lines
☒	☐	Palaeontology and archaeology
☒	☐	Animals and other organisms
☒	☐	Human research participants
☒	☐	Clinical data
☒	☐	Dual use research of concern

Methods

n/a	Involved in the study	
☒	☐	ChIP-seq
☒	☐	Flow cytometry
☒	☐	MRI-based neuroimaging

Antibodies

Antibodies used	Antibodies used in this study were anti -FLAG M2-Peroxidase (HRP), Clone M2-A8592 (Sigma Aldrich) product number A8592 (dilution 1:3000), anti-HA High affinity (clone 3F10) product number 11867431001 (Roche) (dilution 1:2000), PGK1 Monoclonal (22C5D8) product number 459250 (Invitrogen) (dilution 1:10,000), DPM1 monoclonal (5C5A7) product number A6429 (Thermo Fisher Scientific)(dilution 1:10,000). Polyclonal anti-Sei1 (rabbit) (dilution 1:3000), anti-Ldb16 (rabbit) (dilution 1:3000) raised in house.

Validation

Anti-Flag-M2-Peroxidase validation can be found on line at https://www.sigmaaldrich.com/GB/en/product/sigma/f3165?context=product.
Anti-HA High Affinity validation can be found on line at https://www.sigmaaldrich.com/GB/en/product/roche/roahaha?context=product
PGK1 validation can be found on line at https://www.thermofisher.com/antibody/product/PGK1-Antibody-clone-22C5D8-Monoclonal/459250
DPM1 validation can be found on line at https://www.thermofisher.com/antibody/product/DPM1-Antibody-clone-5C5A7-Monoclonal/A-6429
Both Sei1 and Ldb16 polyclonal antibodies were previously validated in "Teixeira, V. et al. Regulation of lipid droplets by metabolically controlled Ldo isoforms. J. Cell Biol. 217, 127–138 (2018)".
